# Notes and News

**Published:** 1892-05-21

**Authors:** 


### Notes and News.

COLLECTED AND COMMUNICATED.

Contrary to expectation, the Lords' Committee sat again last week to take evidence on uniformity in hospital accounts. Mr. Thomas Ryan, of St. Mary's Hospital, was examined as to the action of the Committee of Secretaries of which he was Chairman, and the Committee adjourned.

Mr. Henry Irving writes: "At the request of the Council of the North London Consumption Hospital, I have willingly consented to preside at the annual festival dinner, to be held in the Whitehall Rooms, Hotel Metropole, on Saturday, 28th May. The North London Consumption Hospital receives patients from all parts of the kingdom. Three-fourths of the sum necessary for its structural enlargement has already been contributed, and it is hoped that the result of this appeal may complete the necessary sum. Such a charity must commend itself to all, for there is scarcely a family in the kingdom which has not felt in some form the ravages of this fell disease, and there are few, indeed, who have not through it lost a friend. I shall be happy to acknowledge gratefully any subscriptions sent through to me."

The stable-door is usually shut when the horse has fled; but there are exceptions to all rules, as was recently illustrated by the action of the jury at the Coroner's court at Poplar. An inquest was held on the body of a woman who had died suddenly at a public-house. The parish doctor's assistant found the woman dead on arr ving at the house in answer to a summons. The parish doctor himself attended the inquest, and replied in the negative to the question of one of the jurors as to whether his assistant was qualified. A rider was added to the verdict of the jury that medical men in Poplar should employ qualified assistants only. This doubtless is very sound policy, and considering that the unqualified assistant is not permitted to attend inquests, the objection to them holds good if on this ground alone. Still Poplar strikes us as in advance of its generation, for it desires rather to prevent than cure what might one day cause a serious evil or grave scandal.

It will be a feature of interest at the Chicago Exhibition if Mrs. Ernest Hart's scheme for an Irish Industrial Village is realised. A committee has been formed, consisting of Lady Aberdeen, Mrs. Ernest Hart, and Mr. Peter White, to carry out the plans under the auspices of the Irish Industries Association, and Mr. Peter White sails this week for America to make the necessary arrangements. The project, it is stated, has already aroused considerable interest in the States.

An amusing and satisfactory testimony of what has been done through the aid of the Provident Surgical Appliance Society was told by Dr. Bond at a recent meeting. He stated that a young countrywoman, who has been supplied by the society with an artificial nose of the *retroussé* sort, had, it seems, been so greatly improved in appearance that she has already had more than one offer of marriage. After this may we not expect some fair damsels will be applying for the society's aid under "false pretences?"

Another example has been added to the many of the insufficient precaution taken against accidents from fire at juvenile theatrical performances. Fortunately, at the last case on record no fatality occurred. The pampas grass carried by some children during a performance at a board school caught fire in a gas jet, and as usual a panic ensued amongst both audience and performers, and had the children's dresses been of inflammable mate ial, doubtless some injury would have been done. Surely, where such performances as this are held under official supervision, as was the case we quote, no possibility of a conflagration should be permitted to exist.

The International copyright is all very well, but the McKinley tariff must destroy international courtesy between authors and journalists so far as the United States of America is concerned. If an English book of importance is sent direct to an American journal for review, carriage paid, it may be rejected by the intended recipient because of the heavy import dues charged. In this case the book would be returned to the author, accompanied by a demand for payment for all outstanding charges. In a recent instance which has come to our notice, an important weekly review received volumes of important works from an English publisher. The publisher of this paper sent in a bill for the McKinley dues, and seemed altogether to resent the impertinence of the English publisher and authors for presenting costly works to American journals. The English publishers having requested the American journalist, failing payment of the dues, to forward the books to one of the great American libraries, where they would be welcomed, to his astonishment has received a request that other volumes may be sent to the same paper. It has long been a matter of astonishment to English journalists that the press of America should sit down quietly under a system of tariffs and regulations which interfere with international knowledge and progress, and is an impediment to the interchange of courtesies which are encouraged by the Governments of every other country in the world, except the United States of America.

